# Successful treatment of retinopathy of prematurity in oculocutaneous albinism with *OCA2* variants: a case report and review of literature

**DOI:** 10.1186/s12887-024-04864-2

**Published:** 2024-06-10

**Authors:** Xiao-Yu Zheng, Ding-Wen Wu, Lan Yu, Zheng-Yan Zhao

**Affiliations:** 1 Department of Ophthalmology, Children’s Hospital, Zhejiang University School of Medicine, National Clinical Research Center for Child Health, Hangzhou, China; 2 Department of Genetics and Metabolism, Children’s Hospital, Zhejiang University School of Medicine, National Clinical Research Center for Child Health, Hangzhou, China; 3 Children’s Hospital, Zhejiang University School of Medicine, National Clinical Research Center for Child Health, Hangzhou, China; 4 Department of Child Health Care, Children’s Hospital, Zhejiang University School of Medicine, National Clinical Research Center for Child Health, No. 3333 Binsheng Road, Hangzhou, 310052 Zhejiang Province People’s Republic of China

**Keywords:** Retinopathy of prematurity, Oculocutaneous albinism, OCA2, Laser photocoagulation, Anti-vascular endothelial growth factor

## Abstract

**Background:**

Oculocutaneous albinism (OCA) is a group of autosomal recessive hereditary disorders that affect melanin biosynthesis, resulting in abnormalities in hair, skin, and eyes. Retinopathy of prematurity (ROP) is a proliferative retinopathy mainly observed in premature infants with low birth weight and early gestational age, but it can also affect full-term infants or children with normal weight, particularly in developing countries. The coexistence of ROP and OCA is rare. There is limited documentation regarding treatment approaches, with few studies reporting positive outcomes with laser treatment due to the absence of melanin pigment. This study discusses the treatment challenges in a female infant diagnosed with ROP and OCA, and underscores the importance of genetic analysis in guiding therapeutic decisions for this rare comorbid condition.

**Case presentation:**

The study presents a case of ROP occurring concurrently with OCA. Genetic testing revealed two variants, c.727C > T (p.R243C) and c.1832 T > C (p.L611P), in the *OCA2* gene, inherited from the patient's mother and father, respectively. The identified mutations were consistent with a diagnosis of OCA2, classified as a subtype of OCA. The patient initially received intravitreal anti-vascular endothelial growth factor (anti-VEGF) injection, followed by laser photocoagulation therapy for a recurrent event. A favorable outcome was observed during the 2-month follow-up period.

**Conclusions:**

The co-occurrence of ROP and OCA is a rare phenomenon, and this is the first recorded case in the Chinese population. The current case supports the use of laser as the primary treatment modality for ROP in OCA2 patients with partial pigmentation impairment. Furthermore, genetic analysis can aid in predicting the effectiveness of laser photocoagulation in this patient population.

## Background

Oculocutaneous albinism (OCA) is a group of autosomal recessive inherited disorders characterized by the lack or absence of melanin pigment synthesis in the hair, skin, and eyes. The estimated worldwide prevalence of OCA is approximately 1 in 17,000, while in China, it is 1 in 18,000 [1, 2]. OCA can lead to severe visual impairment in early childhood, including vision loss, photophobia, nystagmus, and strabismus [3].

Retinopathy of prematurity (ROP) is a proliferative retinopathy that predominantly affects preterm infants or low birth weight children, but it can also occur in mature infants. The co-occurrence of ROP and OCA is rare, and the literature on the treatment outcomes of ROP in OCA patients is limited [4–7]. Intravitreal anti-vascular endothelial growth factor (anti-VEGF) therapy and laser photocoagulation have been reported in treating ROP in OCA patients, however, there is no definite consensus on the choice of therapy [4, 6].

Herein, we present the case of a Chinese infant girl diagnosed with ROP and OCA, who relapsed after anti-VEGF injection and, ultimately, achieved resolution with laser photocoagulation.

## Case presentation

A female infant, born prematurely at 31 weeks of gestation with a birth weight of 1560 g, was screened for ROP at 40 weeks postmenstrual age (PMA). The infant had a complex medical history, including respiratory distress, bronchopulmonary dysplasia, anemia, atrial septal defect and OCA. She reported no remarkable family history of OCA or eye disease.

**Fig. 1:**
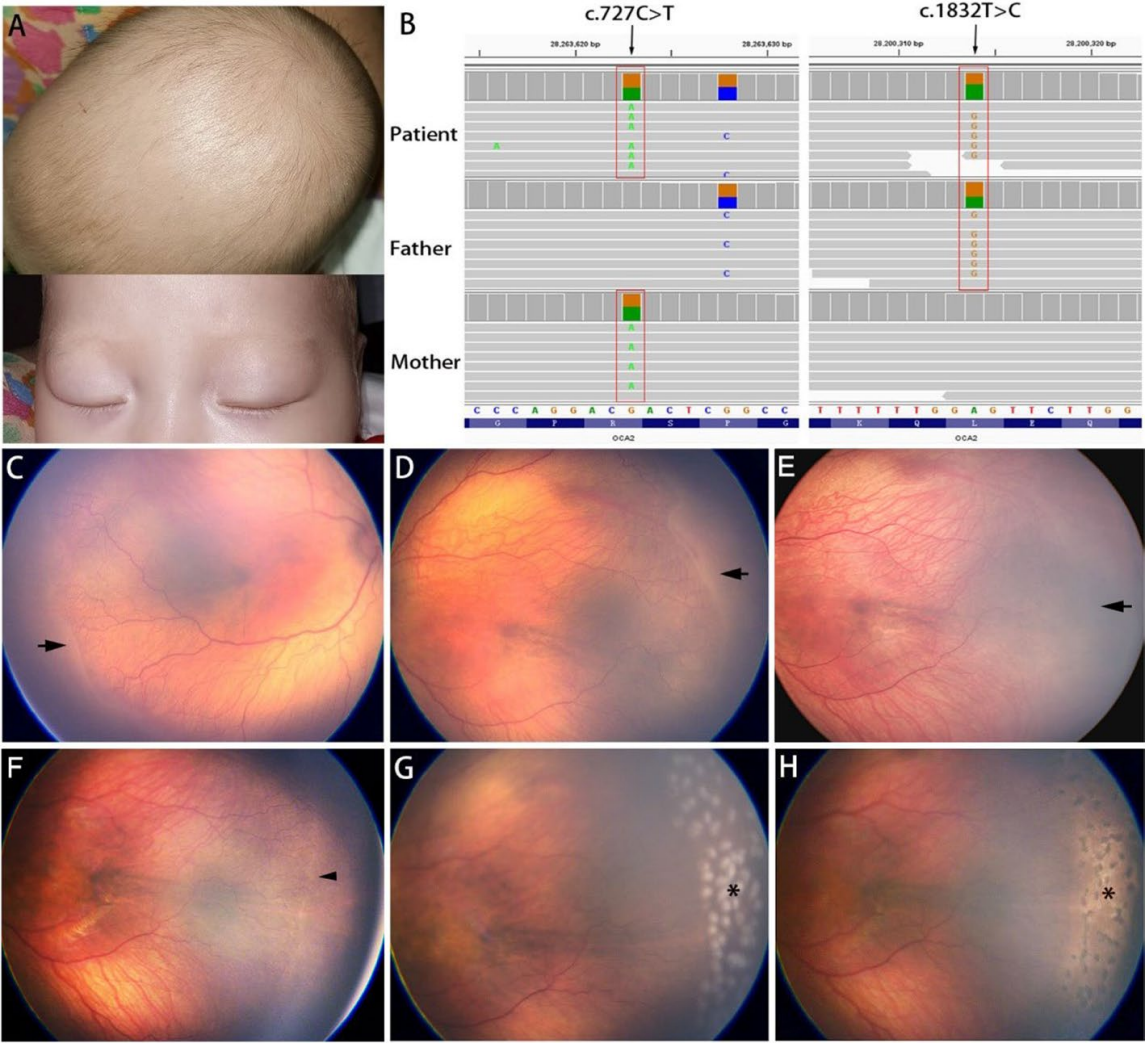
**A** Patient's visible oculocutaneous albinism (OCA) symptoms: skin, eyebrows, and hair color. **B** Visualization of the *OCA2* mutation using Binary Alignment/Map (BAM) format files in Integrative Genomics Viewer (IGV). The mutations found in this study are marked in red rectangles. Note: *OCA2* is encoded by the reverse complement sequence, and c.722C > T is a polymorphic site. **C, D** Fundus images of the right and left eyes at 40 weeks postmenstrual age (PMA), showing zone II stage 3 retinopathy of prematurity (ROP) with pre-plus disease. The black arrow indicates the ridge. **E** Week 1 post-injection: regression of ridge (black arrow). **F **Reactivated stage 2 ROP in zone II, manifested by a faint ridge (arrowhead). **G** Day 1 post-photocoagulation: distinct laser spots (asterisk). **H** Week 1 post-photocoagulation: pigment proliferation within laser scar (asterisk)

Prior to presentation, whole-exome sequencing identified compound heterozygous mutations in the *OCA2* gene in this infant (Fig. 1 C). Specifically, the mother carried the c.727C > T (p.R243C) heterozygous variant, and the father carried the c.1832 T > C (p.L611P) pathogenic variant.

The clinical examination revealed golden eyebrows, brown hair, brown eyelashes, and relatively pale skin (Fig. 1 A, B). The ocular examination revealed light brown iris in both eyes. The fundus assessment was conducted using wide-field fundus photography (Panocam, Visunex, Suzhou, China) and indirect ophthalmoscopy with a 20D noncontact lens. The fundus displayed visible choroidal vessels on an orange background, which is attributed to retinal pigment epithelium (RPE) hypopigmentation. Arterial tortuosity and venous dilation of the posterior retinal vessels indicated pre-plus disease. A proliferative fibrovascular membrane was observed along the ridge at the junction of vascular and avascular retina in zone II of both eyes (Fig. 1 D). A diagnosis of bilateral symmetrical zone II, stage 3, pre-plus disease was made. This condition carries a high risk of progression to type 1 prethreshold disease, for which intervention, in the form of anti-VEGF injections or laser ablation, is required to prevent vision loss [8].

Due to the potential for vascularization into the peripheral avascular retina post-injection and the concerns regarding the efficacy of laser treatment in OCA patients, the infant’s parents opted for anti-VEGF injection over laser treatment to control disease progression. At 41 weeks PMA, intravitreal ranibizumab (0.25 mg/0.025 mL, Novartis, Switzerland) was injected into both eyes under topical anesthesia. During the first week of follow-up, the ridges and pre-plus diseases gradually resolved (Fig. 1 E).

Regular retinal screening was scheduled every two weeks. At 50 weeks PMA, the reappearance of ridges, which indicated the recurrence or reactivation of ROP, was noted. Over the subsequent 4 weeks, despite the ridges not being very prominent and the absence of plus disease, we observed a gradual widening of the ridges, accompanied by tortuous peripheral vasculature and the appearance of a few neovascular buds along the ridges. These subtle changes in the fundus, which were difficult to see due to poor contrast, indicated the progression of ROP. The patient was diagnosed with reactivated stage 2 ROP in zone II (Fig. 1 F).

Due to logistical challenges posed by the patient and her family who lived in a very remote area, a proactive approach was planned to manage the recurrence. We hypothesized that repeated anti-VEGF injections would result in a second recurrence of ROP, considering the limited extent of retinal vascularization into the peripheral avascular retina following previous injection treatment. Upon re-evaluating the findings of the genetic testing, we speculated that the child might respond positively to laser treatment due to partial pigmentation impairment in OCA2 type. As the recurrence not meeting type 1 prethreshold ROP, laser treatment was pursued more as prophylaxis for the peripheral avascular retina and the development of ROP, rather than a needed treatment for the disease activity.

**Fig. 2:**
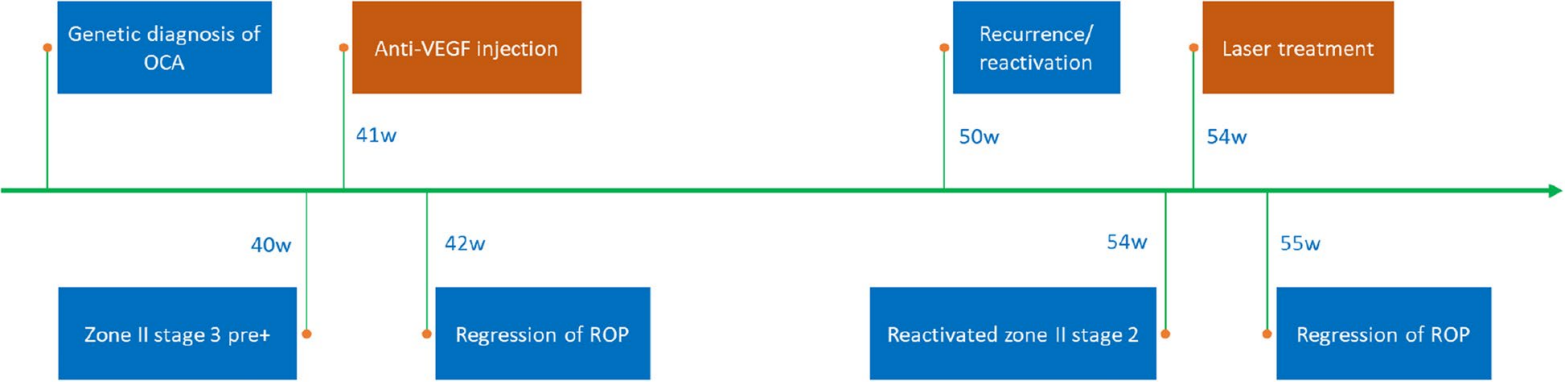
Timeline of the clinical history. w: postmenstrual age (PMA) in weeks

At 54 weeks PMA, laser photocoagulation was performed in both eyes using an Argon green laser (532 nm, YHM-05-DUA, Anywave Technologies, Inc. Beijing, China) under general anesthesia. It was challenging to identify the ridge and ora serrata due to poor contrast in the fundus and retinal vessel blanching under scleral indentation. However, surprisingly, retinal ablation showed strong laser uptake (Fig. 1 G) when parameters of 100 mW power, 300 ms duration, and 300 ms interval were used. Approximately 200 laser spots were applied to each eye, aiming to cover the entire avascular retina. Subsequent follow-up revealed complete regression of the ridge within the following week, with rapid pigmentation in the laser scars (Fig. 1 H). During the 2-month follow-up, ROP regression with no recurrence was observed. The timeline of diagnoses and treatments was presented in Fig. 2.

## Discussion and conclusions

The co-occurrence of ROP with OCA is a rare clinical scenario that requires prompt intervention, especially when type 1 prethreshold ROP is diagnosed. Intravitreal anti-VEGF therapy has emerged as a promising treatment due to its rapid onset of effect, despite potential systemic side effects and long-term safety concerns. Nonetheless, laser photocoagulation remains the gold standard treatment for type 1 ROP and is often preferred for managing reactivated ROP. Although some studies suggest the use of anti-VEGF injections for ROP in OCA patients [5, 7], the reactivation of ROP following anti-VEGF injection in our case prompted us to consider laser treatment. To the best of our knowledge, this is the first documented case of ROP in a Chinese patient with OCA successfully treated by laser photocoagulation.

The therapeutic effects of laser photocoagulation rely on the thermal denaturation of RPE, hemoglobin within the choroid, and the retinal tissue itself. The variability in treatment response is mainly attributed to the presence of RPE melanin, which serves as the primary chromophore within the fundus.

Although OCA patients lack fundus pigmentation, their response to laser therapy has been inconsistent. The majority of the studies indicate treatment failures, with no visible laser marks despite using higher energy settings during surgery [5, 7, 9, 10]. Very few studies have reported cases of successful outcomes. Chandra et al. [4] reported a case of an infant with zone II stage 3 pre-plus disease who underwent laser treatment at 32 weeks PMA. However, no clear laser reaction was observed during the treatment despite increasing the laser power and duration, and the laser scars were barely discernible until the third week postoperatively. Similarly, Gangwe et al. [6] reported a case of aggressive ROP (AROP) in a girl who received diode laser therapy at 33 weeks PMA. Despite increasing the laser power to as high as 500 mW, the laser response remained delayed and very mild during the procedure. AROP did not regress until the sixth week of follow-up. Furthermore, for some other patients with albinism requiring laser treatment, Sinha et al. [11] and Mansour et al. [12] reported a limited success rate of only 50% when using endolaser during pars plana vitrectomy surgery. In contrast to the above studies, the present case exhibited an immediate and favorable response to laser treatment via indirect ophthalmoscopy, followed by a rapid resolution of ROP post-treatment.

In clinical practice, it is often necessary to adjust laser parameters during laser therapy based on the response observed in previous laser spots. Overexposure may lead to undesirable complications, such as patient discomfort, Bruch's membrane rupture, unpredictable scarring, proliferative vitreoretinopathy (PVR) formation, micro-scotoma development, and potential vision impairment [12]. It is important to be cautious about directing too much energy towards the retina, especially in OCA patients with impaired pigmentation. The observation of pigmentation in various areas of the body, especially the fundus, may provide an assessment of the severity of OCA. However, genetic testing, if available, would offer a more reliable method for evaluating the degree of fundus pigmentation and predicting the laser parameters required to achieve a desired outcome.

Genetic testing can be used to classify OCA into different subtypes. OCA1A is characterized by the lifelong absence of melanin production. Laser therapy may not be a suitable option for these patients. On the other hand, OCA1B, OCA2, OCA3, and OCA4 may exhibit varying degrees for melanin accumulation over time [13]. The prevalence of causative genes and OCA subtypes varies significantly across different populations [3]. Mutation analysis within Chinese families primarily identifies OCA1 and OCA2, which range from 22.2% to 88.9% and from 4.8% to 77.8%, respectively [14, 15]. The mutation identified in our patient, specifically c.1832 T > C within the *OCA2* gene (MIM 203200), corresponds to OCA2 phenotype. Compared to other subtypes, OCA2 exhibits a higher likelihood of dark fundus pigmentation [16]. Our report is the first to demonstrate the effectiveness of laser therapy in this specific subtype of OCA. Based on the limited extent of areas requiring laser treatment in this patient (zone II, involving four contiguous clock-hour sectors), we covered all the avascular areas with approximately 200 laser spots in each eye. The number of laser spots was comparable to that in the previous report [6].

To improve the efficiency and safety of laser therapy for patients with OCA, there are some tips that can be followed. Firstly, using a 20-diopter (20D) lens instead of a 28D lens can enhance visualization by magnifying lesions. Secondly, gentle force should be applied when compressing the sclera to minimize retinal blanching. Thirdly, prioritizing laser ablation along the ora serrata and the ridge can provide a clear boundary of the avascular area, thereby preventing skip areas.

In conclusion, the co-occurrence of ROP and OCA is a rare phenomenon. The study supports the use of laser photocoagulation as the primary treatment for OCA patients with clinical signs of partial pigmentation and/or favorable genetic diagnosis. The importance of genetic testing is emphasized in guiding treatment options and predicting outcomes.

## Data Availability

All data generated or analyzed during this study are included in this article.

## Abbreviations

OCA: Oculocutaneous albinism
PMA: Postmenstrual age
PVR: Proliferative vitreoretinopathy
ROP: Retinopathy of prematurity
RPE: Retinal pigment epithelium
VEGF: Vascular endothelial growth factor
